# Acute myeloid leukemia with bipotential erythroid and megakaryocytic differentiation: a case series and literature review

**DOI:** 10.1007/s12308-025-00678-y

**Published:** 2025-12-18

**Authors:** Li-Wei Liu, Shanelle J. De Lancy, Stephanie N. Hurwitz

**Affiliations:** 1 Department of Pathology and Laboratory Medicine, Indiana University School of Medicine, Indianapolis, IN USA; 2 Melvin and Bren Simon Comprehensive Cancer Center, Indianapolis, IN USA

**Keywords:** Acute myeloid leukemia, Megakaryocyte-erythroid progenitor, TP53, JAK1, JAK2

## Abstract

**Background:**

Acute myeloid leukemia with erythroid and megakaryocytic differentiation (AML-EMD) is a rare and aggressive presentation of AML characterized by overlapping morphologic, immunophenotypic, and genetic features of erythroid and megakaryocytic lineages. The classification, pathogenesis, and clinical behavior of this entity remain poorly defined.

**Methods:**

We report a series of three patients in conjunction with a systematic review of reported cases in published literature with AML-EMD.

**Results:**

Cytogenetic and molecular studies revealed frequent abnormalities including complex karyotypes, *TP53* mutations, and *JAK1/2* mutations. Clinically, patients demonstrated a poor-risk profile.

**Conclusions:**

Collective phenotypic and genetic features suggest that AML-EMD represents a high-risk subgroup of either *TP53*-mutated AML or AML with myelodysplasia-related genetics, likely reflecting leukemic transformation from multipotent progenitors retaining erythro-megakaryocytic potential. Despite shared biologic features, current classification systems for AML-EMD are diagnostically incongruent. Recognition of this entity will allow for consistent subclassification, prognostication, and future studies aimed at defining its molecular underpinnings and therapeutic vulnerabilities.

## Introduction

Acute myeloid leukemia with erythroid and megakaryocytic differentiation (AML-EMD) is a rare entity characterized by morphologic and immunohistochemical features of both erythroid and megakaryocytic lineages. The phenotypic description “erythroid and megakaryocytic differentiation” is most often added as a qualifier to myeloid neoplasms defined in the 5th edition of the World Health Organization Classification of Tumours of Haematopoietic and Lymphoid Tissues (WHO-HAEM5) and 2022 International Consensus Classification (ICC), as AML-EMD is not an independent category in either diagnostic guideline [1, 2]. AML-EMD can occur as a primary disease or secondary to myelodysplastic syndrome or anti-neoplastic therapy [3]. The majority of reported cases are associated with complex karyotypes, with a significant subset driven by a combination of JAK/STAT pathway and *TP53* mutations [3–9]. Although rare, the co-expression of erythroid and megakaryocytic lineage markers in neoplastic cells points to a likely cell-of-origin in megakaryocyte-erythroid progenitors (MEP). Here, we present the clinical and pathological presentation of 3 cases of AML-EMD and a review of other reported cases of AML-EMD, shedding light on common pathogenic features of this rare presentation.

## Materials and methods

Three cases of AML-EMD were identified at the University of Pennsylvania and Indiana University. Dual differentiation was defined as having co-expression of at least two erythroid (CD71, glycophorin A, CD117, E-cadherin) and megakaryocytic lineage markers (CD61, Von Willebrand factor, CD42b) in malignant blasts. Peripheral blood, bone marrow aspirate smears, core biopsies, immunohistochemistry stains, flow cytometry, conventional karyotype, and targeted mutational analysis performed at the time were reviewed, along with additional clinical and laboratory findings. Additional reported cases in the literature were identified through extensive searches through PubMed and Google Scholar databases.

## Results

### Case presentations

We identified three elderly male patients (ages 69–74 years) who presented with cytopenias and bone marrow (BM) findings consistent with acute myeloid leukemia (AML) showing dual erythroid and megakaryocytic differentiation. Clinical and laboratory features are summarized in Table 1.

**Table 1:** Clinical and laboratory data of patients at diagnosis

	Case 1	Case 2	Case 3
Age	69	74	70
Sex	M	M	M
Prior diagnosis	IgG kappa multiple myeloma, post-autoHSCT	IgA lambda monoclonal gammopathy	Myelodysplastic syndrome
CBC			
Hemoglobin (g/dL)	6.3	8.8	7
MCV (fL)	90	91	81
WBC (10^3/µL)	3.6	8.4	3.2
Platelets (10^3/µL)	17	11	50
Circulating blasts (%)	1	1	0
Bone marrow			
Aspirate blasts (%)	50–60	< 1	15
Biopsy blasts (%)	70	50	25
Fibrosis	Grade 2–3	Grade 2–3	Grade 1–2
Blast immunophenotype			
CD45	Dim +	Dim, partial +	Dim +
CD117	+	+	+
E-cadherin	+	+	+
Glycophorin A	+	NT	NT
CD71	NT	+	+
CD61	+	+	+
CD34	-	-	Subset +
MPO	-	-	-
TdT	-	-	-

*autoHSCT* autologous hematopoietic stem cell transplant

Two patients had concurrent plasma cell neoplasms (Patient 1: history of multiple myeloma with an autologous hematopoietic stem cell transplant 4 years prior and low-level residual disease at the time of current presentation; Patient 2: monoclonal gammopathy of undetermined significance). The third patient had a reported history of high-risk myelodysplastic syndrome (MDS) with del(5q), del(7q), and *DNMT3A*, *TET2*, and *TP53* mutations. All three patients presented with anemia and thrombocytopenia; circulating blasts were rare or absent.

**Fig. 1:**
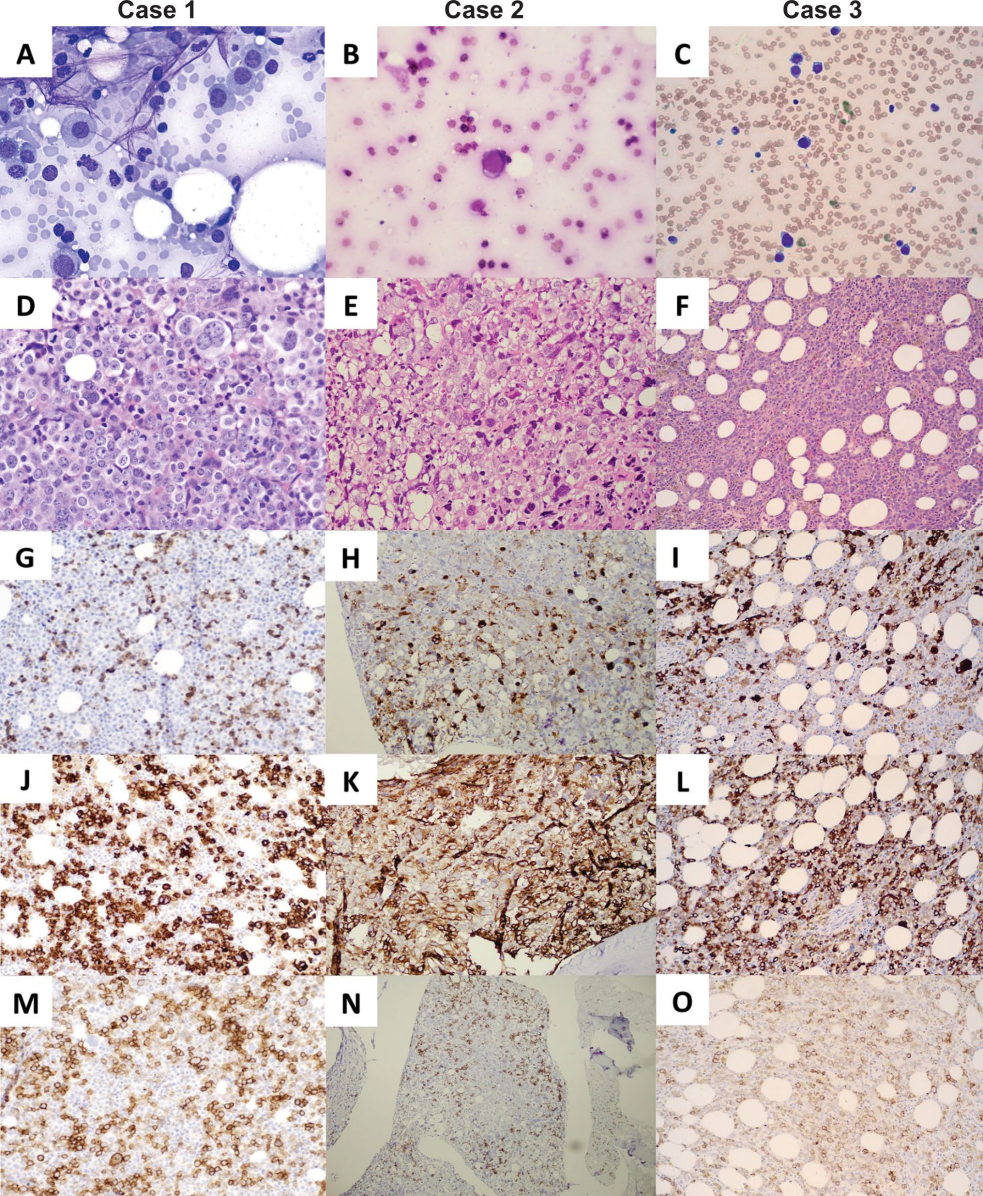
Morphologic and immunophenotypic bone marrow features of AML-EMD. Representative bone marrow (**A**–**C**) aspirate smears showing sparse blasts and (**D**–**F**) hypercellular, fibrotic core biopsies with sheets of blasts and megakaryocytic atypia. Immunohistochemistry staining of blasts showing at least partial positivity of (**G**–**I**) CD61, **J** glycophorin A, **K**–**L** CD71, and (**M**–**O**) E-cadherin

BM aspirates were limited in two of three cases due to significant marrow fibrosis. Aspirate smears and biopsy touch preparations revealed large, atypical cells with blastoid morphology, fine chromatin, prominent nucleoli, and scant to abundant basophilic cytoplasm with vacuolization and occasional cytoplasmic blebbing (Fig. 1). Biopsies showed markedly hypercellular marrows (70–> 95%) with increased reticulin fibrosis and sheets or clusters of immature cells, often with multinucleation. Two cases showed prominent megakaryocytic dysplasia.

By immunohistochemistry, neoplastic cells in all cases demonstrated partial or co-expression (> 10%) of erythroid (E-cadherin, glycophorin A, CD71), megakaryocytic (CD61, CD42b), and immature cell markers (CD117, dim CD45). Myeloperoxidase, CD34, TdT, and lymphoid or plasma cell antigens were negative. Adding to the diagnostic challenge, blasts were not detected by flow cytometric analyses in two of three cases, likely due to significant marrow fibrosis and possible lysis during processing.

Cytogenetic and targeted mutational analyses revealed complex karyotypes in two patients and pathogenic *TP53* mutations in all three cases (Table 2). Additional pathogenic variants included *DNMT3A*, *TET2*, *ASXL1*, *JAK2*, and *SH2B3*. All three patients were treated with AML-directed therapy (decitabine/venetoclax or liposomal cytarabine/daunorubicin).

**Table 2:** Genetic profiles and diagnostic differences in published cases of AML-EMD

Reference	Age (y)	Cytogenetic profile	Mutational profile	WHO-HAEM5	ICC 2022
Case 1	69	46,Y,dic(?X;13)t(?X;13)(q28;p13)ins(X;?)(q24;?)add(13)(q22),add(3)(p12),add(7)(p22),add(8)(p11.2),der(14;21)(q10;q10),add(17)(p13),add(18)(q23),+mar,inc[2]/46,XY[5]	*TP53* R306* (VAF 38%) *TP53* A74Pfs*49 (VAF 8%)	AML, myelodysplasia-related, post-cytotoxic therapy	AML with mutated *TP53*, therapy-related
Case 2	74	NP	*TP53* C176* (VAF 68.9%) *JAK2* V617F (VAF 22.9%) *SH2B3* L178Qfs*88 (VAF 63.7%)	Acute erythroid leukemia/acute megakaryoblastic leukemia	AML with mutated *TP53*
Case 3	70	42~43,XY,add(1)(q21),del(1)(q12),add(4)(q?21),−5,−7,ins(8;?)(q13;?),−11,−13,−15−16,add(19)(p13.3),add(19)(p13.3),−21,add(21)(q22),−22,+1~9mar[cp21]/46,XY[8]	*TP53* Y220C (VAF 17%) *ASXL1* G647Efs*15 (VAF 4%) *DNMT3A* R882H (VAF 31%) *TET2* A1341Cfs*3 (VAF 5%)	AML, myelodysplasia-related	AML with mutated *TP53*, progressing from MDS
Daniels, 2007 [7]	78	Complex karyotypes, all variations contain del(5) and 2 marker chromosomes	NR	AML, myelodysplasia-related	AML with myelodysplasia-related cytogenetic abnormalities
Jang, 2014 [5]	14	47,XY,+8,t(12;20)(p13;q13.1)[18]/46,XY[2] *RUNX1T1* amplification	NR	AML, post-cytotoxic therapy	AML with myelodysplasia-related cytogenetic abnormalities, therapy-related
Wang, 2015 [3]	30	Complex karyotype	NR	AML associated with Schwachman-Diamond syndrome	AML associated with Schwachman-Diamond syndrome, Progressing From MDS
Xiao, 2018 (1) [4]	> 60	Amplification of 13q31-q32, 19q13-qter, and 21q22, Del(4q), Del(11p), add(18p) CN-LOH of 17p *ERG* amplification *RUNX1* amplification	*JAK1* V658I (VAF 4%) *TP53* Y236C (14%), *TYK2* V603M (48%) *TET2* P1012L (52%)	AML, myelodysplasia-related	AML with mutated *TP53*
Xiao, 2018 (2) [4]	> 60	69,+1,+1,+2,+3,+6,+6,+7,+8,+8,+8,+8,+10,+12,+14,+14,+15,+17,+18,+19,+19,+21,+21,+22 CN-LOH of chromosomes 2p, 4q, and 17p	*JAK2* V617F (VAF 36%) *TP53* V272M (VAF 75%) *TET2* S1838Cfs*2 (VAF 43%) *DNMT3A* P627Qfs*24 (VAF 72%)	AML, myelodysplasia-related	AML with mutated *TP53*
Chernak, 2020 [9]	57	NP	*JAK2* V617F *TP53* Q167* *MGA* I2281K *KMT2C* I811V *PRSS3* X124_Splice	AML, myelodysplasia-related	AML with mutated *TP53*, progressing from MDS/MPN
Thakral, 2021 [6]	< 1	48,XX,+8,+21c[18]/47,XX,+21c[2]	*GATA1* mutation *RAD21* mutation *SUZ12* mutation	Myeloid leukemia associated with Down syndrome	Myeloid leukemia associated with Down syndrome
Chan, 2022 [8]	72	52~53,XY,+1,add(1)(p36.1),+2,add(3)(q29),+4,+6,−7,+8,add(8)(q24),−11,−15,add(19)(p13),+20,+21,+4mar,inc[3]/46,XY [17]	NR	AML, myelodysplasia-related	AML with myelodysplasia-related cytogenetic abnormalities

*NR* not reported, *NP* not performed, *CN-LOH* copy neutral loss of heterozygosity, *AML* acute myeloid leukemia, *MDS* myelodysplastic syndrome, *MPN* myeloproliferative neoplasm

### Additional reported cases in the literature

In addition to our three patients, we were able to identify eight cases, 2 pediatric and 6 adults, with AML-EMD after an extensive search on the PubMed and Google Scholar databases (Table 2) [3–9]. The majority of AML-EMD occurred de novo*,* with a minority occurring in a treatment-related setting. All adult cases presented with complex karyotypes, with a significant subset harboring *TP53* mutations, three of which were accompanied by *JAK1* or *JAK2* mutations [3, 4, 7, 9]. One pediatric case occurred in the setting of post-treatment T-lymphoblastic lymphoma; the other case arose in the setting of Down syndrome with transient abnormal myelopoiesis (TAM) [5, 6]. The recommended diagnostic classification, as defined in the ICC and WHO-HAEM5, of each case is listed in Table 2.

## Discussion

The 2022 ICC recognizes a category of AML with mutated *TP53*, which is defined by a *TP53* mutation with a VAF ≥ 10%. The diagnosis of AML with *TP53* mutation takes precedence over AML with myelodysplasia-related cytogenetic anomalies (AML-MRC) due to the poor prognosis *TP53* mutations convey [10, 11]. The majority of identified cases of AML-EMD fall into AML with mutated *TP53* (when mutational analysis was performed) or AML-MRC, with the exception of two cases occurring in patients with Down syndrome and Schwachman-Diamond syndrome respectively [3, 6].

In contrast, AML with *TP53* mutations is not an independent classification in the WHO-HAEM5 [11]. Given their complex karyotypes, the majority of reported AML-EMD fall into the category of AML, myelodysplasia-related (AML-MR). The classification of AML-EMD secondary to germline predisposition follows a similar pattern as the ICC. For cases that fail to meet criteria for genetically defined AML, including those with insufficient material for testing as in patient 2 of this study, the WHO-HAEM5 recommends classification based on differentiation. However, a category to include overlapping erythroid and megakaryocytic differentiation does not currently exist.

Overall, the genetic landscape of AML-EMD is complex. As mentioned, *TP53* mutations are particularly enriched in these cases (6/7 tested patients) [4, 9]. AML-EMD with *TP53* mutations appear to follow the trend of poor outcomes in other AMLs with *TP53* mutations [11]. However, this is unlikely to be the only contributing factor as the dismal outcome was also observed in AML-EMD without identified *TP53* mutations or alterations to chromosome 17p13.1. *JAK1/2* mutations are similarly enriched in AML-EMD. In patient 2, prominent megakaryocytic hyperplasia and dysplasia hinted at the possibility of evolution from an underlying *JAK2*-mutated myeloproliferative neoplasm (MPN). Interestingly, we only observed *JAK1/2* mutations in cases with concomitant *TP53* mutations (3/6 patients). We also note a case of Down syndrome–associated AML-EMD that highlights an alternative GATA1-driven pathogenesis of dual erythroid and megakaryocytic differentiation [6, 12, 13].

Mechanistically, synergistic *Jak2* V617F and loss-of-function *Trp53* mutations have been noted to drive fully penetrant, transplantable AML in a murine model, pointing to a likely mechanism of transformation seen in many post-MPN AML and AML-EMD patients, involving BMP2/SMAD pathway activation [14, 15]. *Jak2* and *Trp53* biallelic inactivation together lead to an expansion of aberrant megakaryocytic-erythroid progenitors (MEPs), consistent with bipotential lineage markers seen in the identified cases in this study. A recent computational tool mapping AML patient samples onto a single-cell reference atlas of human hematopoiesis has also shed light on distinct differentiation states of genetically defined AML [16]. Recurrent genetic alterations in AML-EMD, including complex karyotypes and pathogenic mutations in *TP53*, *JAK2*, and *GATA1* mutations found in AML-EMD, were associated with early erythroid and MEP/megakaryocytic progenitor differentiation states.

Importantly, identification of this unique differentiation state has relevance to clinical management. Similar to that described in *TP53*-mutated AML, erythroid/megakaryocytic differentiation may confer resistance to the BCL-2 inhibitor, venetoclax, through BCL-XL overexpression [17–20]. Recognizing that many of these cases harbor *JAK2* mutations, combined BCL-XL/JAK inhibition may be a possible therapeutic avenue [19]. Given the significant biologic and prognostic overlap with *TP53*-mutated AML, a diagnosis of MDS/AML-EMD should certainly prompt immediate testing for *TP53* loss-of-function mutations. If testing is not able to be performed, we suggest that pathologic diagnosis should note the presence of dual erythroid and megakaryocytic differentiation. With the exception of Down syndrome–related cases, clinical consideration may be given to treating presumptively as a *TP53*-like AML. In the future, updated classification frameworks may allow for more unambiguous terminology to define these rare cases.

Overall, these findings suggest that cases of reported AML-EMD likely represent biologically similar diseases, with diagnostic challenges and incongruent classifications by current WHO and ICC standards. Further studies are needed to examine leukemic transformation of AML-EMD lacking *JAK2* and *TP53* mutations. A deeper understanding of both the genetic and differentiation states of AML-MRD will complement existing strategies to improve diagnostic classification, risk stratification, and drug response prediction.

## Data Availability

No datasets were generated or analysed during the current study.
